# Supplementary material for: Connectivity Is All You Need: Inferring Neuronal Types with NTAC
Source: bioRxiv. 2025 Jun 11:2025.06.11.659184. Preprint. [Version 1] doi: 10.1101/2025.06.11.659184 (PMC12259112; doi:10.1101/2025.06.11.659184)
Supplement: 1 [file NIHPP2025.06.11.659184V1-supplement-1.pdf]

bioRxiv preprint doi: https://doi.org/10.1101/2025.06.11.659184; this version posted June 11, 2025. The copyright holder for this preprint (which was not certified by peer review) is the author/funder, who has granted bioRxiv a license to display the preprint in perpetuity. It is made available under aCC-BY 4.0 International license.

# Supplemental data

## Datasets Statistics

| | Full Brain (F) | Visual System (F/M) | OL Intrinsic (F/M) | Central Brain (F) | Ventral Nerve Cord (M) |
|---|---|---|---|---|---|
| # Neurons | 139,255 | F: 94,721<br>M: 52,445 | 86,048<br>45,046 | 39,732 | 23,655 |
| # Connections | 5,608,727 | F: 4,425,266<br>M: 6,484,936 | 3,204,551<br>4,716,893 | 1,017,756 | 5,305,638 |
| # Types | 8,537 | F: 740<br>M: 793 | 230<br>234 | 7,829 | 4,351 |

bioRxiv preprint doi: https://doi.org/10.1101/2025.06.11.659184; this version posted June 11, 2025. The copyright holder for this preprint (which was not certified by peer review) is the author/funder, who has granted bioRxiv a license to display the preprint in perpetuity. It is made available under aCC-BY 4.0 International license.

**Table SD1.** The male visual system contains only the right hand side, the female contains both. OL Intrinsic is a subset of the visual system, which itself is a subset of the full brain. Central brain is a subset of the full brain. Full brain and the ventral nerve cord together form the CNS, while neck neurons belong to both. See illustration in Fig. 1.

# Analysis of inaccuracies

Taking a deeper look into cases where NTAC struggles on the columnar[3] neurons we observe that the inaccuracies occur on the boundaries (T4*, T5* neuron types). Using 5% labeled neurons yields 80% accuracy, compared to >95% accuracy on complete visual system.

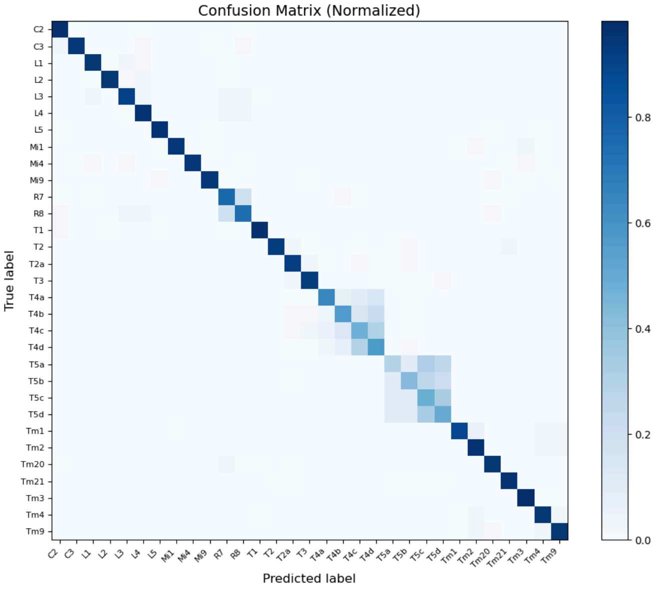


**Figure SD1.** Confusion matrix for the columnar types. The most problematic classes are T4 and T5 subtypes, and they are misclassified among themselves when the dataset at hand does not include downstream projection neurons to differentiate between them.

[3] Cell types in the FlyWire Optic Lobes which consist of at least 700 instances per hemisphere: C2, C3, L1, L2, L3, L4, L5, Mi1, Mi4, Mi9, T1, T2, T4a, T4b, T4c, T4d, Tm1, Tm2, Tm4, Tm9, Tm20, R7, R8, T2a, Tm3, T3, Tm21, T5c, T5b, T5a, T5d.

bioRxiv preprint doi: https://doi.org/10.1101/2025.06.11.659184; this version posted June 11, 2025. The copyright holder for this preprint (which was not certified by peer review) is the author/funder, who has granted bioRxiv a license to display the preprint in perpetuity. It is made available under aCC-BY 4.0 International license.

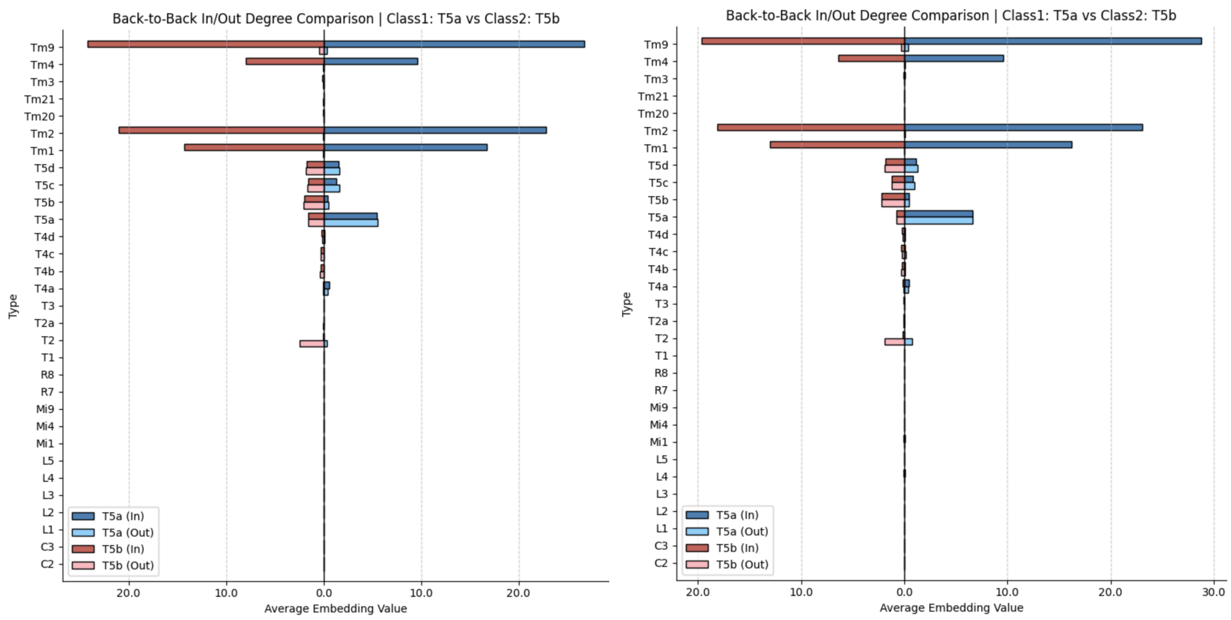


**Figure SD2.** The average neighborhood histogram (the “fingerprints”) of T5a compared to T5b. On the left we use the ground truth partition and on the right we use the partition returned by NTAC. Plots show the average neighborhood value for a specific type over all neurons in the class. This implies that using only the topological features of the columnar neurons, the T5 types are nearly impossible to distinguish. However, when classifying a larger dataset (not just the columnar cells in isolation), the algorithm behaves much better because their downstream partners (visual projection neurons) carry the information to differentiate them. Indeed, running NTAC on all intrinsic neurons in the optic lobe we get near perfect accuracy (99%) on both T4 and T5 type neurons.

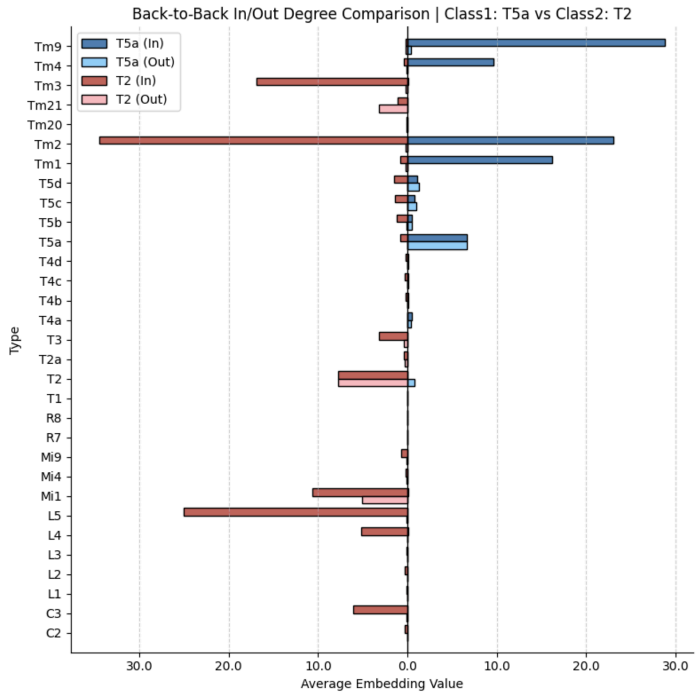


**Figure SD3.** As a sanity check we compare T5a and T2: we can see that there is a significant difference.

bioRxiv preprint doi: https://doi.org/10.1101/2025.06.11.659184; this version posted June 11, 2025. The copyright holder for this preprint (which was not certified by peer review) is the author/funder, who has granted bioRxiv a license to display the preprint in perpetuity. It is made available under aCC-BY 4.0 International license.

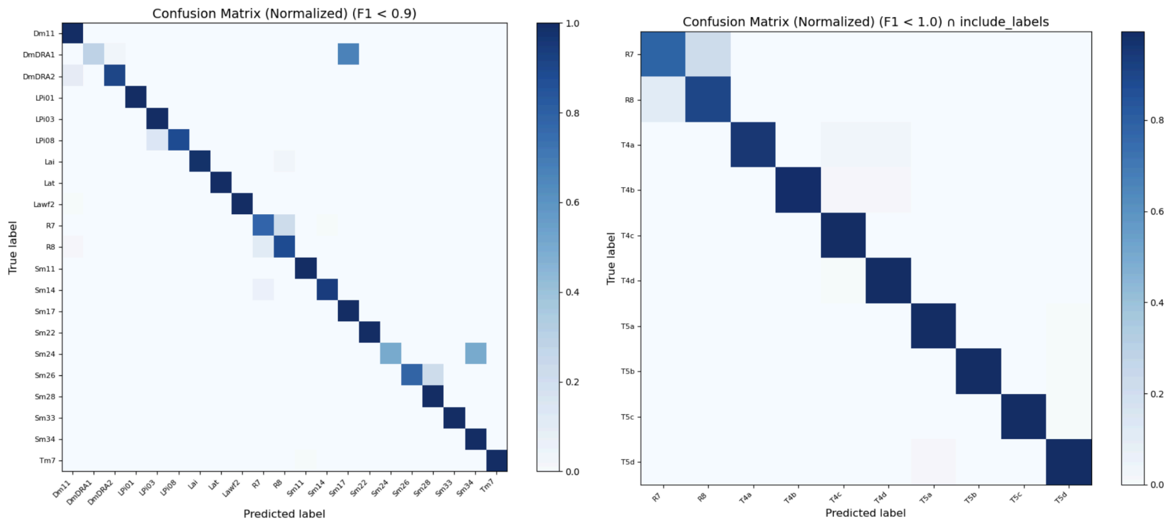


**Figure SD4.** The confusion matrices for the intrinsic dataset. As the number of labels here is much larger, we plot one confusion matrix for all types which have an F-score below 0.9, and another matrix for the problematic types from the columnar dataset. We also note that the confusion between R7 and R8 persists even in the larger dataset, and this is expected since in the FlyWire connectome the reconstruction quality near the edge of the lamina was affected by a partial severing of the lamina from the medulla, which reduced the fidelity of photoreceptor reconstructions, including R1–R6 and R7/R8, and led to lower synapse attachment rates in this region (Dorkenwald et al. 2024).

bioRxiv preprint doi: https://doi.org/10.1101/2025.06.11.659184; this version posted June 11, 2025. The copyright holder for this preprint (which was not certified by peer review) is the author/funder, who has granted bioRxiv a license to display the preprint in perpetuity. It is made available under aCC-BY 4.0 International license.

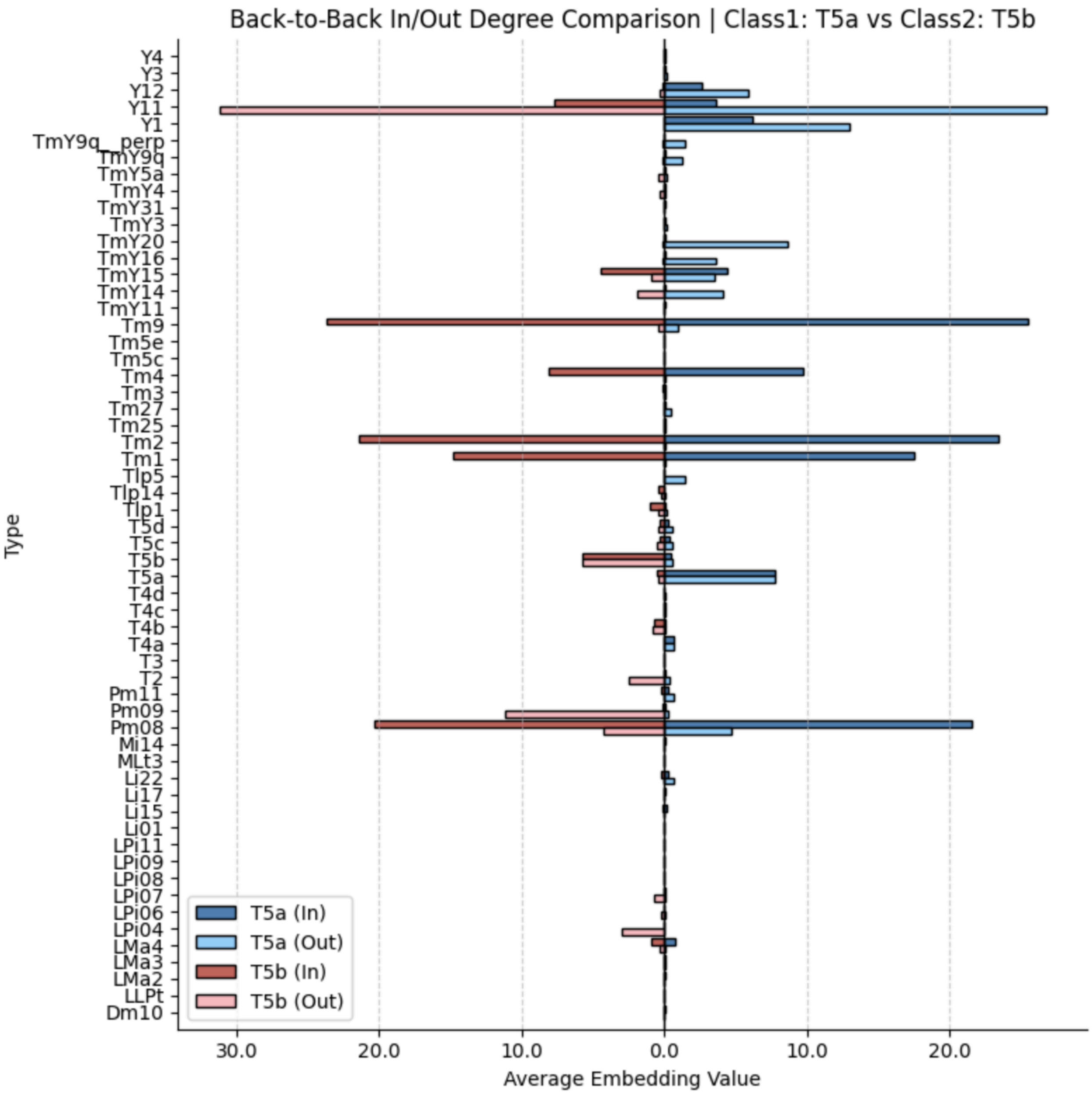


**Figure SD5.** The histograms of T5a and T5b in the larger dataset. As the embeddings are very large now, let us only keep entries where both values exceed a threshold of 0.05 so that we can generate a meaningful diagram. We can now see that the two classes can be separated using topology alone.
